# Enhancing Sustainable Analytical Chemistry in Liquid Chromatography: Guideline for Transferring Classical High-Performance Liquid Chromatography and Ultra-High-Pressure Liquid Chromatography Methods into Greener, Bluer, and Whiter Methods

**DOI:** 10.3390/molecules29133205

**Published:** 2024-07-05

**Authors:** Sami El Deeb

**Affiliations:** Institute of Medicinal and Pharmaceutical Chemistry, Technische Universität Braunschweig, 38106 Braunschweig, Germany; s.eldeeb@tu-braunschweig.de; Tel.: +49-531-391-7301

**Keywords:** green chemistry, green analytical chemistry, blue analytical chemistry, white analytical chemistry, sustainable analytical chemistry, sustainability, sustainable development, sustainability guidelines, solvent selection, liquid chromatography

## Abstract

This review is dedicated to sustainable practices in liquid chromatography. HPLC and UHPLC methods contribute significantly to routine analytical techniques. Therefore, the transfer of classical liquid chromatographic methods into sustainable ones is of utmost importance in moving toward sustainable development goals. Among other principles to render a liquid chromatographic method green, the substitution of the organic solvent component in the mobile phase with a greener one received great attention. This review concentrates on choosing the best alternative green organic solvent to replace the classical solvent in the mobile phase for easy, rapid transfer to a more sustainable normal phase or reversed-phase liquid chromatography. The main focus of this review will be on describing the transfer of non-green to green and white chromatographic methods in an effort to elevate sustainability best practices in analytical chemistry. The greenness properties and greenness ranking, in addition to the chromatographic suitability of seventeen organic solvents for liquid chromatography, are mentioned to have a clear insight into the issue of rapidly choosing the appropriate solvent to transfer a classical HPLC or UHPLC method into a more sustainable one. A simple guide is proposed for making the liquid chromatographic method more sustainable.

## 1. Introduction

**Figure 1:**
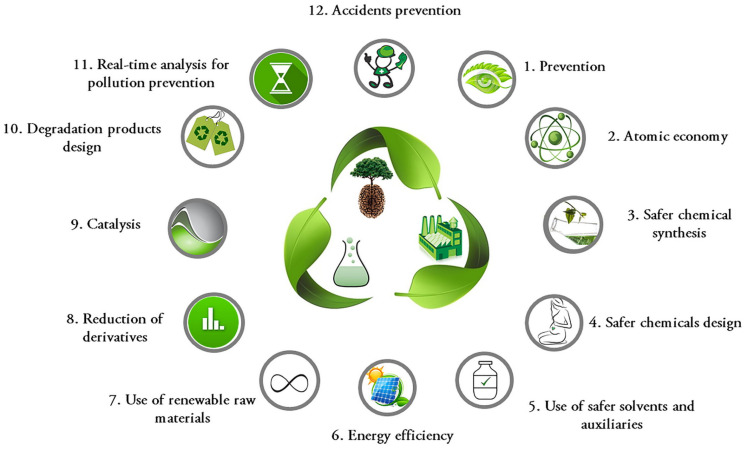
Twelve principles of green chemistry proposed by Anastas and Warner are reprinted with permission from reference [3].

Different scientific disciplines are considering the United Nations Sustainable Development Goals for 2030 with their environmental, social, and economic pillars in their activities [1]. Analysts are raising awareness to move toward more sustainable practices in chemistry. Analytical chemistry is a unique player in environmental and health sustainability. In one way, analytical chemistry acts as a tool to test the toxicity level in different media, and in another way, it utilizes chemicals that can be hazardous to the environment and humans. At the end of the 20th century, the concept of green chemistry was introduced by Anastas and Warner [2], with 12 principles of green chemistry to reduce health and environmental footprints presented nicely by B.A. de Marco et al. [3], as shown in Figure 1.

**Figure 2:**
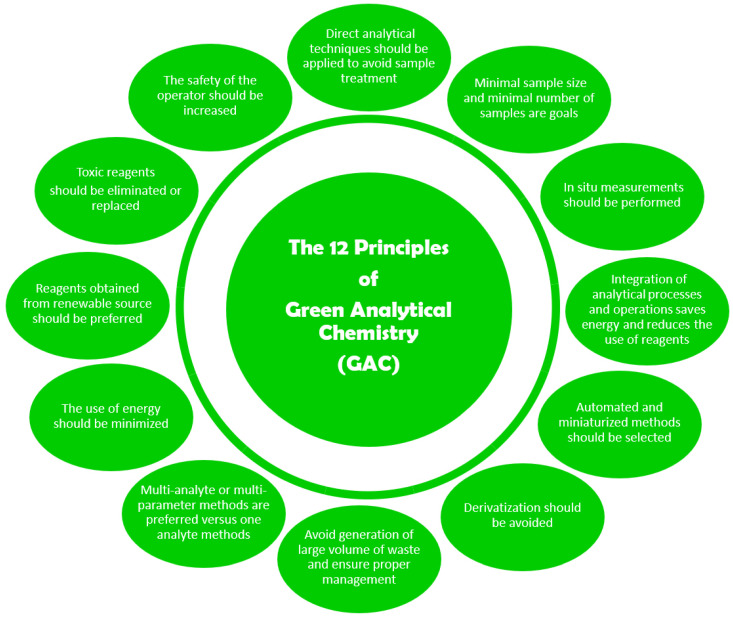
Representative diagram showing the 12 principles of green analytical chemistry was created by the author for the principles proposed by Galuszka et al. [4].

Twelve principles of green analytical chemistry (GAC) were later proposed and published by Galuszka et al. [4]. They were adapted for analytical chemistry and represented a basic guideline for going green in analysis, as shown in Figure 2.

Unlike techniques such as capillary electrophoresis [5,6], supercritical fluid chromatography [7], and sensor-based analytical techniques [8] that are quite green from a sustainable point of view, liquid chromatography utilizes larger amounts of organic solvent in the mobile phase [9], generating huge amounts of toxic waste and emitting relatively large amounts of carbon dioxide that affect global warming by slowing the production of ozone in the lower stratosphere. Moreover, the extensive use of volatile organic compounds (VOC) also acts as ozone-depleting chemicals to further contribute to the global warming potential [10].

In analytical practice, normal phase and HILIC phase chromatography utilize more toxic non-polar organic solvents than reversed phase chromatography and are thus considered less green, with more possibilities for toxic solvent accumulation. However, reversed phase chromatography is more commonly applied in routine analysis, so consideration of all is important. Analytical and preparative liquid chromatographic methods are integral parts of analytical separations, including chiral separations, identifications, analytical characterizations, and determinations of chemicals. They contribute strongly to pharmaceutical research, from the drug discovery and development process to routine quality control. The Environmental Protection Agency (EPA) [11] and the International Organization for Standardization (ISO) promote transferring classical liquid chromatographic methods to green analytical chemistry [12].

Organic solvent usage and waste production account for more than half of the greening of a classical method. Therefore, the primary focus when transforming a reference LC method with a toxic organic solvent in the mobile phase into a greener method is the replacement of the toxic solvent with a greener one and, whenever possible, a reduction in solvent consumption and, thus, waste generation. In practice, using a green solvent to replace a toxic one is an important principle, among others, for easy transfer to green analytical methods. Greening the liquid chromatographic methods can, among other aspects, be easily enhanced by replacing the classical solvent with a greener alternative. The use of biodegradable solvents can further consolidate the reduction in waste generation. Whenever not already existing in the classical method and if available, this could be combined with the replacement of the separation column with a higher-performance one. Militarization of chromatographic columns also plays a vital role in method greening. The use of monolithic or core–shell columns with improved performance and thus shorter length and internal diameter significantly reduces the analysis time, saves solvent and energy, and enhances the greenness of the method. The same is also true for 3 µm and sub-2 µm particle columns, where smaller particle sizes are reflected by a larger surface area and better separation performance. When necessary, a UHPLC instrument is applied to resist the high backpressure associated with the use of small particles [13].

The use of shorter columns with faster analysis time would also reduce the instrumental energy consumption per run, which would decrease the carbon footprint and further increase the greenness of the method. In principle, less energy consumption related to both solvent and instrument lowers carbon emissions and enhances the greenness of the method. This would contribute to total carbon dioxide emissions. It has been shown that analytical laboratories emit about 22% of the amount of carbon dioxide emissions associated with petrol cars per day [14]. Therefore, HPLC and UHPLC instruments are regarded as energy-intensive instrumental techniques associated with high carbon footprints. This can be minimized by depending on renewable energy sources such as solar power and wind energy or by reducing the analysis time to decrease energy consumption [15]. The energy consumption of HPLC and UHPLC instruments differs based on vendor and version; instruments with low energy consumption are desired. LC vendor companies should consider further investments to improve their instrumentations in terms of reduced energy demand for power savings to contribute to a lower carbon footprint and render their instruments less polluting. On the other hand, companies, as well as research and educational laboratories, should aim to implement newer, more efficient LC instruments with lower energy consumption to reduce the carbon footprint associated with analysis. Scientists suggest including energy consumption and carbon footprints in the validation criteria of new analytical methods [14].

Sustainable analytical chemistry should be globally adopted shortly. Pharmacopeia should implement newer, alternative, greener methods and modernize traditional LC analytical methods to elevate the sustainability of analytical chemistry. Therefore, scientists should suggest more alternative green LC separation methods to replace traditional non-green methods. This refinement is mandatory shortly to improve the analytical sustainability of pharmacopeial methods. However, the total switch to sustainable methods should start earlier in the global pharmaceutical industry and research laboratories. Until they are officially included as method validation criteria, chemical and pharmaceutical companies should consider method sustainability in their laboratory guidelines.

**Figure 3:**
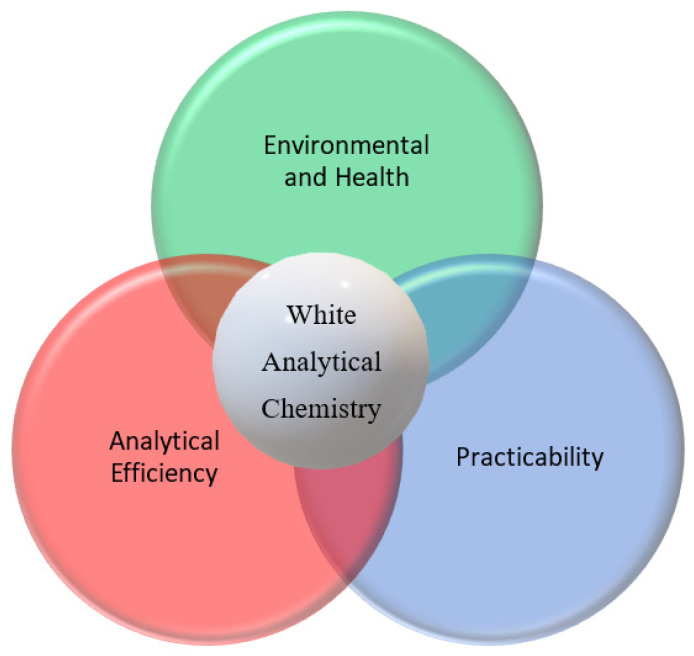
Simple diagram represents the three components of white analytical chemistry.

In the past few years, further terms have been popularized, extending the consideration beyond green analytical chemistry. The term blue analytical chemistry is concerned with ensuring the practicability of the green analytical method in terms of ease of use and cost-effectiveness [16]. In many cases, greening the analytical method would be at the expense of its performance. Thus, the method will become greener while the analytical performance will be compromised, and this might affect its intended application. It is necessary to maintain an adequate level of method performance (e.g., precision, sensitivity) when greening it to ensure that the method can fulfill its purpose. Therefore, a further advancement toward better sustainable analytical chemistry has been considered by Nowak et al. [17] in 2021, who came up with a new approach beyond green analytical chemistry named white analytical chemistry (WAC) as an extension of red, green, and blue principles. WAC considers beyond the environmental aspects of the analytical method its analytical and practical aspects. Under the term WAC, the three main components, namely method greenness with a green color component, method analytical efficiency with a red color component, and method practicability with a blue color component, are included, as represented in Figure 3. The three components are weighted to give an overall white color strength, representing the sustainability percentage of the method [17,18].

Several reviews have been published on green and beyond analytical chemistry; however, they did not present a clear solvent selection guide or method transfer guide to shift a traditional LC method based on toxic organic solvents to a more sustainable method [19,20,21,22,23,24,25,26].

This review aims to give insight into green solvent selection for chromatographic application while considering environmental, health, and chromatographic suitability and compatibility aspects. This should encourage analysts in industrial companies, research institutes, and the educational sector to rapidly transfer their well-established conventional LC into sustainable LC methods and eliminate the use of toxic organic solvents in the mobile phase that are harmful to the environment and humans. The awareness of solvent and instrument energy consumption should also encourage the use of high separation efficiency columns that can allow fast analysis with reduced energy consumption and a lower carbon footprint. The paper also aims to discourage the use of intensively power-consuming old liquid chromatographic instrumentation.

## 2. Solvent Selection

### 2.1. Solvent Selection Guidelines

**Figure 4:**
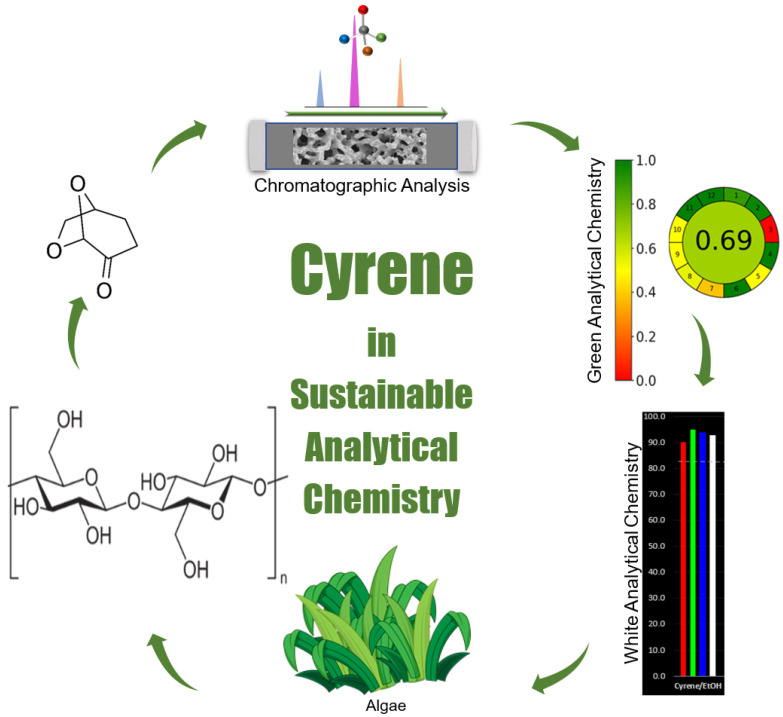
Representative diagram showing the use of Cyrene in chromatography was created by the author.

Several solvent selection guidelines, like those of Pfizer, GSK, and Sanofi, and the combined approach of the three (Pfizer, GSK, and Sanofi), AstraZeneca, the ETH Zurich approach, the Rowan University approach, the ACS GCI solvent selection guide, the International Council for Harmonization (ICH) Q3C (R8) guidelines, and the CHEM21 guide, were published for ranking and rating organic solvents according to their environmental, health, and safety (EHS) problems, considering similar or sometimes different criteria where solvents appeared sometimes with different ranking priorities [27,28,29,30]. All probably lack the emphasis on sustainable solvents for liquid chromatographic analysis. Solvent selection guidelines to rank solvents based on their greenness are mainly established with an orientation to the use of the solvent in synthesis and might be biased when considering their use for chromatographic analysis. For instance, according to the CHEM21 solvent selection guideline, the environmental (E) profile for dihydrolevoglucosenone (Cyrene) is scored 7 and assigned as a problematic solvent because of the high boiling point and thus the difficulty of evaporating when used in synthesis. However, this high boiling point is considered an advantage when thinking about its suitability for chromatographic analysis because it makes it easy and inexpensive to recycle and allows the possibility of running heated and superheated liquid chromatography. Therefore, the ranking for chromatography can be reversed depending on the suitability of liquid chromatography. An ideal, disadvantage-free, and completely sustainable organic solvent for LC analysis is still unavailable. Based on the EHS environmental, health, and safety index, a favorable green solvent for chromatographic analysis is one that can be produced from biomass routes with low energy and low cost compared to petrochemical routes and one that is also biodegradable. Biobased solvents should, whenever possible, be integrated into liquid chromatographic analysis to enhance sustainability. For instance, Cyrene is an organic solvent that is available as a bio-based chemical from renewable feedstock and has shown promising potential for use as an organic solvent in chromatography [31], as represented in Figure 4.

### 2.2. G-Score

**Figure 5:**
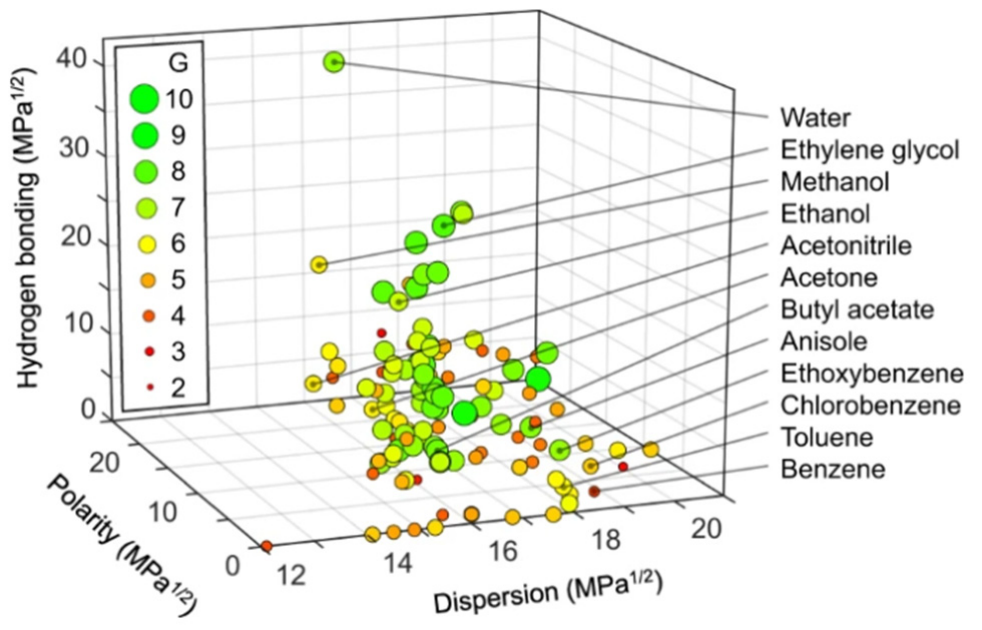
Graphic presentation of the G-score of the solvents in the Hansen space.

Hansen space for solvent selection evaluates solvent greening based on Hansen Solubility Parameters (HSP) [32]. The score of solvent systems with their GSK greenness (G) Score is available freely as a web tool in the Hansen Space with a G-score graph under http://green-solvent-tool.herokuapp.com (accessed on 1 June 2024), as shown in Figure 5.

The highest G-score value is 10 to indicate a fully eco-friendly solvent. In practice, most common liquid chromatography green solvents have a G-score between 6 and 8. Solvents with a G-score below 6 are not preferred in green chromatography. Propylene carbonate still has the highest G-score as an LC green organic solvent, with a value of 8.8. However, propylene carbonate suffers, among other disadvantages like pressure fluctuation and high viscosity, from low water solubility and thus miscibility with the aqueous mobile phase portion. This could be improved by the mixed solvent concept by adding another more soluble green co-eluent, ethanol (in a tertiary mobile phase system), to improve the solubility [33]. The author of this manuscript calculated the G-score value of Cyrene, which has been recently proposed as a green organic solvent for chromatographic application by El Deeb et al. [31].

The G-score of Cyrene is not readily available in the free web of Hansen space but has been calculated according to the following equations [34], considering health (H), safety (S), environment (E), and waste disposal (W) categories of the GSK’s Solvent Sustainability Guide shown in Table 1 [35]:

$$G=\sqrt[{4}]{H\times S\times E\times W}$$

where the H category includes the subcategories health hazard (HH) and exposure potential (EP) and can be calculated using the solvent values in the GSK solvent guide (in Table 1) according to the flowing equation:

$$H=\sqrt{\mathrm{HH}\times \mathrm{EP}}$$

and S category represents the safety category that includes the subcategories flammability and explosion (F&E) and reactivity and stability (R&S) and can be calculated using the solvent values in the GSK solvent guide (in Table 1) according to the following equation:

$$S=\sqrt{F\&E\times R\&S}$$

and E category represents the environmental category with subcategories air impact (Air) and aqueous impact (Aqua) can be calculated using the solvent values in the GSK solvent guide (in Table 1) according to the following equation:

$$E=\sqrt{\mathrm{Air}\times \mathrm{Aqua}}$$

and W category to represent waste disposal with the subcategories incineration (I), recycling (R), bio treatment (BT), and volatile organic compounds (VOC) can be calculated using the solvent values in the GSK solvent guide (in Table 1) according to the following equation:

$$W=\sqrt[{4}]{I\times R\times \mathrm{BT}\times \mathrm{VOC}}$$

If unavailable, one can calculate the G-score of any organic solvent based on the information mentioned in the GSK solvent sustainability guide according to the above equation.

### 2.3. Relative Hazard

The relative hazard indicates the chemical hazard of the substance (in this case, the organic solvent) relative to the chemical hazard of chloroform (CH_sub_/CH_CHCl3_). A relative hazard could be used to indicate the degree of chemical risk associated with the use of a solvent; thus, a smaller value indicates a greener solvent. The chemical hazard of chloroform (CH_CHCl3_) equals 5.75. A simple model called weight hazard number (WHN) can be used to calculate the chemical hazard of the substance. According to the WHN model, the chemical hazard of a substance is calculated based on the following equation:

WHN(CH_sub_) = 1.*N_cat1_* + 0.75.*N_cat2_* + 0.5.*N_cat3_* + 0.25*N_cat4_*

where *N_cat_* is the number of hazards in a given category according to the safety data sheet (SDS) of the substance (solvent).

Values of chemical hazards according to WHN for each of the common solvents are provided in Table 2, either obtained from reference [36] or calculated by the author based on recent SDS category values of each solvent. It is worth noting that the relative hazard can be multiplied by the mass of the substance to give what is referred to as the chloroform-oriented toxicity estimation scale (ChlorTox Scale) based on the following equation to act as an indicator for chemical risk [36].

ChlorTox = CH_sub_/CH_CHCl3_.M_sub_

### 2.4. Consideration of Chromatographic Suitability

When choosing an organic solvent for use as a component in the mobile phase of liquid chromatographic analysis, more information is required about the solvent than its greenness score to judge its suitability for the analysis to substitute the traditional hazardous organic solvent in the reference method. Some factors are not considered in either G-score or relative hazards but are LC-relevant and can play a good role in selecting a green organic solvent for chromatographic applications. These factors include compatibility with the detector, miscibility of the organic solvent with the aqueous phase of the mobile phase, elution power, density, boiling point, and purity.

The primary detection method in HPLC and UHPLC is UV/Vis spectrometry; thus, the transparency of the solvent in this region accounts for its advantages as a green solvent; otherwise, its applicability will be limited to substances that can absorb beyond the ultraviolet (UV)/visible cut-off value of the solvent. Compatibility with other common detection techniques, like mass spectrometry and fluorescence detection, is an advantage. Therefore, the compatibility with the used detectors should be known. Actually, UV transparency is an important limiting factor in the implementation of a new green organic solvent in various chromatographic applications. The narrower the transparency range, the less possibility there is to apply for a wide range of substances that absorb only out of this range or have very weak absorbance within the transparency range that does not fulfill the required sensitivity for the intended application.

It is important to consider the miscibility of the green organic solvent with the aqueous component of the mobile phase when it substitutes the old organic solvent. In cases of very low solubility, a co-eluent may be added in small amounts to improve the solubility. Otherwise, an alternative green solvent should be tried. It is worth noting that solubility is somewhat involved in greenness considerations. In general, low water solubility and a high Log P value indicate high bioaccumulation and aquatic toxicity. It is also important to consider the solving power of the solvent to solubilize analytes, depending on their polarity. The elution power of a newly implemented organic solvent in LC should also be considered, as should its compatibility with different stationary phases for normal and reversed-phase chromatography. Showing a similar selectivity and retention behavior to the toxic organic solvent in replacement would make method transfer easier. A primary impression about the elution power of the new green solvent compared to the old toxic solvent can be expected by comparing the polarity parameter Kamlet–Taft (π*) values [37]. The high density of the organic solvent should be considered in view of the developed back pressure. As mentioned before, the boiling point of the organic solvent for chromatographic application is preferably high to facilitate waste treatment and offer the possibility of high-temperature separation [38].

Green solvents assigned and ranked for synthesis or purification require less purity than solvents for chromatographic analysis, where the presence of impurities as contaminant elements might hinder their application through reactivity with analytes, non-transparency in detection, and fluctuation with a non-smooth baseline. This should be an issue to consider when trying to implement a new green solvent for use in chromatographic analysis. The comprehensive testing of new potential green organic solvents for chromatographic analysis is essential to advance the field of sustainable analytical chemistry.

In Table 2, the author of this manuscript listed the properties and parameters of 17 solvents for normal, HILIC, and reversed-phase liquid chromatography, taking into account suitability parameters for liquid chromatographic analysis in addition to greenness. The suitability requirements should be balanced against the greening requirements to choose the best solvent for the intended application. The values for each solvent are based on the solvent data sheet SDS, G-score, and relative hazard resources. Subject to a future update with more solvents, Table 2 should act as a current collated solvent selection guide for liquid chromatography.

Certain parameters have been particularly mentioned in Table 2 to give a rapid indication of the health and safety of the solvent. For instance, a value lower than 2000 mg/kg of the health measure rat oral LED50 can indicate a harmful solvent [39]. The vapor pressure of the solvent can reflect its volatility and, thus, its ozone-depleting potential. Substances with high vapor pressure will vaporize more readily, as stated by the United States Environmental Protection Agency. A vapor pressure of 10 hPa at 20 °C or more (0.01 kPa at 293.15 K or more) represents a VOC–ozone-affecting solvent [40]. The WHO classified inorganic pollutants as very volatile, volatile, and semi-volatile organic compounds, depending on the boiling point; a low boiling point indicates a more volatile organic compound [41]. The flash point, as a critical measure of flammability, shows the lowest temperature at which the substance can vaporize to form an ignitable mixture. It also gives a rapid indication of solvent safety and should be above 60 °C [42]. A high partition coefficient n-octanol/water (log P value) value of more than 4 indicates high lipophilicity and bioaccumulation potential [43].

### 2.5. Liquid Chromatography Sustainability Guideline

**Chart 1:**
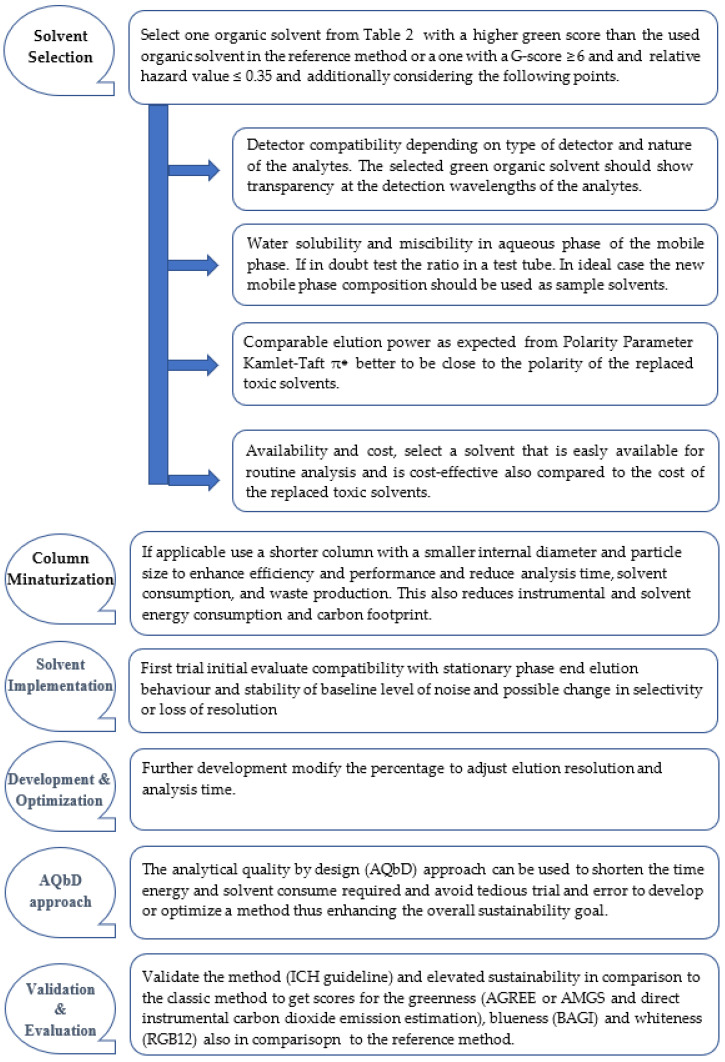
Simple liquid chromatographic sustainability guideline (LCSG) for the transformation of classical to sustainable HPLC or UHPLC methods.

The following chart represents a simple guide to transferring a traditional classical non-green LC method into a greener, more sustainable LC method by organic solvent replacement and, whenever applicable, by changing to a higher separation efficiency column (Chart 1). It is worth noting that greening the sample preparation method, if applicable, using the same green liquid as the sample solvent would further enhance the overall method’s green score. The suggested transformation just concentrates on eliminating the toxic organic solvent through green alternative solvent replacement and possibly smaller columns to reduce solvent consumption, waste production, and analysis time. There is an economic and environmental benefit associated with organic solvent waste reduction in analysis. In ideal cases, organic solvent should be eliminated whenever possible, like in the case of transferring to heated or superheated water chromatography [44]. This could also be applied to high-boiling-point liquids like Cyrene, as suggested by El Deeb et al. [31].

It is worth mentioning that the greener replacement solvent should not be of significantly larger volume than the replaced toxic solvent to avoid increasing the overall use of organic solvent in the method, which negatively impacts the greenness profile. In some cases, green solvents could be worse because of the significantly larger volume required to replace the toxic solvent. The strategy in Chart 1 acts as a greening guideline in Table 2, which would help to subjectively choose a proper green solvent and implement it in the LC method to elevate its analysis sustainability.

## 3. Post-Greening Method Evaluation

It is advisable to assess the greening profile of the developed or transformed green method. The evaluation tools would demonstrate the superiority of a proposed green method over a traditionally reported method.

### 3.1. Greening Evaluation

Many recently developed analytical chromatographic methods fail to meet green analytical criteria. Several evaluation tools have gained significant recognition and acceptance within the analytical chemical society, mostly using friendly shareware software that generates a colored pictogram in some of them with a quantitative numerical value in percentage where 100% reflects full alignment [45,46].

Here are two recommended tools to use in evaluating the greenness of your method. The first is AGREE, which stands for Analytical GREEnness Metric Approach and Software, considering the 12 principles of green analytical chemistry and presented as a pictogram with a score in the middle and green, yellow, and red colors for each segment to indicate the agreement level with the greenness principle. It is easy-to-use, user-friendly software (version 0.5 beta) with a simple, illustrative colored pictogram. It represents a comprehensive, well-recognized tool commonly used to evaluate greenness and compare methods after transformation from classical to green [47,48].

The second is referred to as AMGS and stands for Analytical Method Greenness Score. It is a tool to compare method greenness considering three main issues, namely instrument energy, solvent energy (energy demand associated with solvent production and incineration for disposal), and solvent EHS aspects [49]. It is an open-source spreadsheet calculator and is available online at “https://www.acsgcipr.org/amgs (accessed on 1 June 2024)”. The lower the overall score of AMGS, the greener the method. The detailed scores of greenness percentage will be given for each of the three components: instrument energy, solvent energy, and EHS.

It is worth noting that the carbon footprint associated with the use of an HPLC or UHPLC instrument can be directly calculated according to the following equation to obtain a value for kilograms of carbon dioxide equivalent (carbon footprint) per analysis.

Kg CO_2_ eq = ⅀ Instrument Power (kW). Analysis time (h). Emission factor for electricity (kg CO_2_/kWh)

The reference constant value for the emission factor is 0.247 kg CO_2_/kWh. Instrument power differs depending on the analytical instrument [50].

### 3.2. Blueness Evaluation

The Blue Applicability Grade Index (BAGI) is free software available under “https://Bagi-Index.Anvil.App (accessed on 1 June 2024)”. It involves involving 10 questions with variable choices for each to evaluate the practicability of the method. The software evaluates practicability aspects and ease of application, including the type of analysis, number of elements, analytical technique, sample preparation, number of samples analyzed per hour, reagents, pre-concentration, degree of automation, and amount of sample [16].

### 3.3. Whiteness Evaluation

The whiteness of the method can best be evaluated using the RGB12 tool with the freely available Excel sheet [17] to evaluate the three components, each with four column aspects. The red component has four aspects covering the scope of application: LOD and LOQ, precision, and accuracy. The green component has four aspects: toxicity of the reagent, amount of reagent and waste, consumption of energy and other media, and direct impact. The blue component has four aspects, namely cost-efficiency, time efficiency, sample consumption, the need for advanced instruments, and operational simplicity. By filling in the required data in each component, a graphical presentation of red, green, and blue columns with a white column saturation depending on the relative fill of each component will be presented to indicate the percentage of method whiteness [18].

## 4. Conclusions

Green and white analytical chemistry are currently gaining significant attention to support the general global move toward sustainability. Analysts aim to move toward a more sustainable future in analytical chemistry that can be implemented in routine analytical work. For instance, routine quality control of pharmaceuticals should, in the future, be conducted as sustainable quality control with energy-efficient practices and a minimal environmental burden. Analysis of real pharmaceutical mixtures and bio-analytical applications in drug monitoring and forensic investigations should also be conducted using energy-efficient and cost-effective methods. Currently, applied analytical methods still depend on the use of hazardous organic solvents, which contravenes method greenness. The use of easily available, inexpensive reagents and the simplicity of the method, with the possible elimination of laborious steps such as pre-concentration derivatization or a complex gradient program, should also be considered to support the practicability of the method. A handful of alternative organic solvents for chromatographic elution are demonstrating superiority over routinely used hazardous organic solvents in terms of greenness. It is worth noting that any sustainable analytical method could undergo further optimization to elevate its sustainability profiling without sacrificing practicability or method performance. The article should increase the analytical method’s sustainability awareness. This should enhance sustainable practice in analytical chemistry using HPLC and UHPLC instruments, which are dominant in analysis with cost-effective, energy-efficient, eco-friendly methods that reduce carbon dioxide emissions and minimize waste production. Reducing the carbon footprint and VOC can positively contribute to reducing global warming. The author expects that this paper will provide good insight into the implementation of sustainable analytical chromatography in industrial, research, and educational fields. Sustainable analytical chemistry is currently in increasing practice and will play a crucial role in the near future to maintain sustainability and contribute more to sustainable development.

## Figures and Tables

**Table 1:** GSH of some organic solvents, including dihydrolevoglucosenone (Cyrene), shows the subcategory values required to calculate its G-score [35] *, **.

Classification	Solvent Name	CAS Number	Composite Colour	Boiling Point (°C)	Incineration	Recycling	Biotreatment	VOC Emissions	Aquatic Impact	Air Impact	Health Hazard	Exposure Potential	Flammability and Exlosion	Reactivity and Stability
**Halogenated**	1,2,4-Trichlorobenzene	120-82-179-11-8		214	3	7	7	10	1	9	4	6	9	10
	Chlorobenzene	108-90-7		132	4	9	7	7	2	7	4	4	8	10
	1,2-Dichlorobenezen	95-50-1		180	4	8	6	7	1	6	7	6	8	10
	Trichloroacetonitrile	545-06-2		83	4	8	5	4	3	4	4	3	7	10
	Perfluorotoluene	434-64-0		104	4	4	6	6	1	7	4	4	5	10
	Flurobenzene	462-06-6		85	4	7	5	4	3	4	4	3	4	10
	Perflurocyclic ether	335-6-4		103	4	4	6	6	1	7	4	4	5	10
	Dichloromethane	75-09-2		40	2	10	4	1	8	6	7	4	4	10
	1,2-Dichloromethane	107-06-2		84	2	7	5	5	9	7	1	2	5	10
	Perflurocyclohexane	355-68-0		53	4	9	5	2	4	7	4	2	3	10
	Chloroform	67-66-3		61	3	9	5	3	7	5	4	1	5	10
	Trichloroacetic acid	76-03-9		197	1	4	3	9	2	5	4	6	10	6
	Chloroacetic acid	79-11-8		189	1	4	4	9	6	4	1	5	10	6
	Trifluoroacetic acid	76-05-1		72	1	5	2	4	4	4	4	3	7	6
	Perfluorohexane	355-42-0		57	4	10	5	2	1	7	4	2	4	10
	Carbon tetrachloride	56-23-5		77	3	7	5	4	4	1	4	1	4	10
	2,2,2-Trifluoroethanol	75-89-8		74	1	5	2	4	5	4	1	1	6	9
**Other**	Furfural	98-01-1		162	7	8	8	8	8	4	4	6	9	9
	*N*,*N*-Dimethyldecanamide	14433-76-2		291	6	7	6	10	4	6	10	10	10	10
	Dihydrolevoglucosenone	1087696-49-8		203	4	4	5	10	9	6	4	8	10	10
	*N*,*N*-Dimethyloctanamide	1118-92-9		261	5	6	5	8	7	5	4	6	9	10
	*N*,*N*-Dimethylaniline	121-69-7		194	7	7	6	9	3	4	4	8	9	9
	Acetic anhydride	108-24-7		140	4	6	4	8	8	7	4	4	8	6
	Nitromethane	75-52-5		101	2	1	4	6	6	8	7	5	7	1
	Triethylamine	121-44-8		89	4	4	1	5	8	3	4	3	5	6

* Column heading color key showing cumin color of incineration, recycling, biotreatment and VOC emission components to indicate waste category, light green color of aquatic impact and air impact components to indicate environment category, blue color of health hazard and exposure protection components to indicate human health category, and pink color of flammability & explosion and reactivity & stability components to indicate safety. ** Composite color key showing solvent sustainability issue with dark green to indicate few known issues, yellow to indicate some known issues and red to indicate major known issues.

**Table 2:** Organic solvent greenness and chromatographic suitability selection table *.

Solvent	UV Cut-Off Value (nm)	Water Solubility	Density (g/cm^3^) at 20 °C	Polarity Parameter Kamlet- Taft π *	Partition Coefficient n-Octanol/Water (log *p* Value)	Boiling Point °C	Flash Point °C at 1.013 hPa (c.c.)	Vapor Pressure hPa at 20 °C	LD50 Oral-Rat mg/kg	Composite Color of GSK Solvent Sustainability Guide	Relative Hazard (WHN)	G-Score Hansen Space
Acetone	330	miscible in any proportion	0.79	0.71	−0.23	56.05	−18	240	5800		0.35	5.9
Acetonitrile	190	miscible in any proportion	0.7822	0.75	−0.34	82	2	94.51	469		0.39	5.8
Butanol	215	ca. 77 g/L at 20 °C	0.81	0.47	0.78	117.6	37	6.3	700		0.43	6.7
Chloroform	245	ca. 8 g/L at 20 °C	1.498	0.58	1.97	62	9.7	210	908		1.00	4.4
Cyclohexane	210	ca. 0.1 g/L at 20 °C	0.779	0.00	3.44	83	−20	104	>5000		0.87	5.3
Dihydrolevoglucosenone (Cyrene)	350	ca.52.6 g/L at 20 °C	1.25	0.93	−1.52	227	108	0.28	>2000		0.13	6.9
Ethanol	210	≥1000 g/L at 20 °C	0.81	0.54	−0.31	78	9.7	59	10.470		0.26	6.7
Ethyl acetate	255	ca. 87 g/L at 20 °C	0.894	0.54	0.73	77	−4	97	5620		0.35	6.8
Ethyl lactate		miscible	1.03	0.82	0.70	154	46	2.7	>2000		0.39	6.4
Hexane	195	ca. 0.014 g/L at 20 °C	0.655	−0.04	3.90	69	−22	160	25,000		0.78	4.8
Isopropanol	205	miscible in any proportion	0.786	0.48	0.05	82.4	12	43	5840		0.35	6.5
Methanol	205	1000 g/L at 20 °C—completely miscible	0.7913	0.61	−0.77	64.7	12	128	5628		0.57	5.8
Propanol	210	miscible in any proportion	0.803	0.52	0.25	97	23.5	22	1870		0.39	6.6
Propylene carbonate	220	175 g/L at 25 °C	1.2047	0.9	−0.41	240	132	0.04	>5000		0.13	8.8
Tetrahydrofuran	212	miscible in any proportion	0.883	0.58	0.45	65	−21.2	170	1650		0.61	4.8
Toluene	286	ca. 0.573 g/L at 20 °C	0.867	0.50	2.73	110.6	4.4	29	5580		0.86	6.0
Water	190	Not applied	0.9982	1.28	Not applied	100	Not applied	17.535	≥90,000		Not applied	7.3

* Composite Color of GSK Solvent Sustainability Guide with dark green to indicate few known issues, yellow to in-dicate some known issues and red to indicate major know issues.

## Data Availability

No new data were created or analyzed in this study.
